# A Conversation with Carolyn Pearce

**DOI:** 10.1021/acscentsci.3c01628

**Published:** 2024-01-02

**Authors:** Rachel Brazil

For seven decades, 177 tanks of radioactive waste have lain stagnant under a now-decommissioned nuclear production complex in Hanford, Washington. The site was the home of the first full-scale plutonium production reactor, established in 1943 as part of the Manhattan Project.

**Figure:**
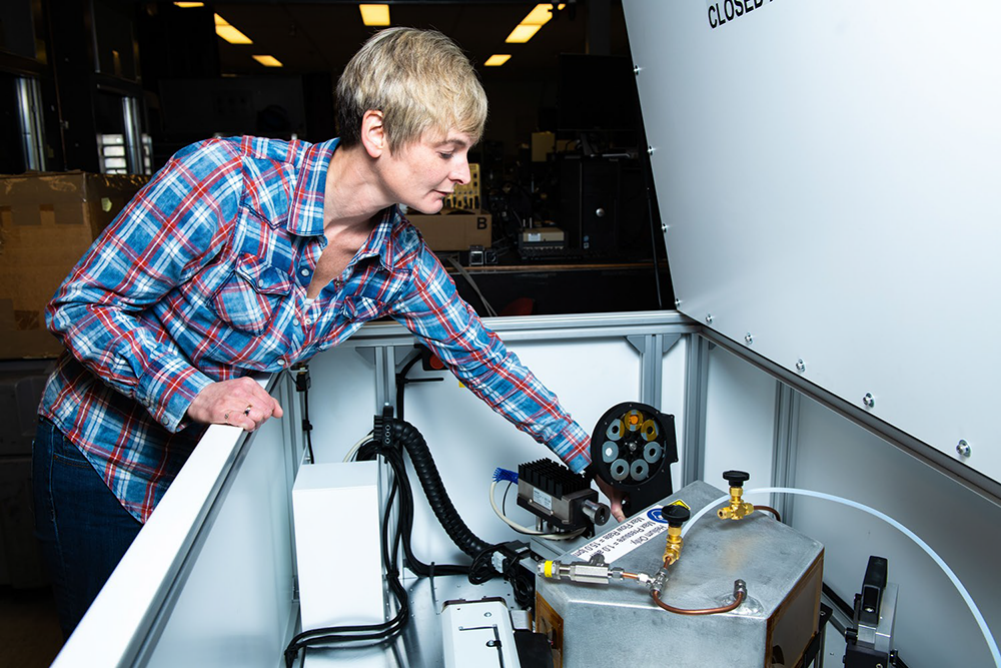
Carolyn Pearce makes adjustments to Pacific Northwest National Laboratory’s benchtop X-ray laboratory, which is used to probe the chemical and electronic state of metal atoms in nuclear waste glasses. Credit: Andrea Starr/Pacific Northwest National Laboratory.

Of the 200 million-plus L of radioactive waste in the leak-prone tanks, only 10% falls into the high-level category: waste with relatively high concentrations of the most radioactive material, such as fission products. The remaining 90% is classified as low-activity waste: salt solutions with lower concentrations of radionuclide contaminants that pose less risk to human health and the environment. But all that waste still needs to be processed and stored safely.

Carolyn Pearce’s team is putting a plan in motion to deal with much of that material. The approach laid out by Pearce, Director of the Interfacial Dynamics in Radioactive Environments and Materials (IDREAM) Energy Frontier Research Center at Pacific Northwest National Laboratory (PNNL), hinges on vitrification, a process used since the 1970s in which nuclear waste is mixed into molten glass and locked away. After a year’s delay due to a problem with the two 270-metric-ton melters, Hanford’s waste vitrification plant—the world’s largest—is testing the process of pouring molten glass into stainless steel containers for long-term storage at the site’s underground integrated disposal facility (IDF).

To reach this milestone and beyond, Pearce’s team has explored the complex chemistry of the waste in Hanford’s tanks to better predict how it will behave as it goes through processing, gets incorporated into glass, and—hopefully—stays put for a millennium or more. Her group carries out experiments and even looks to millennia-old analogs to gain insight into the long-term survival of glass.

Rachel Brazil recently spoke to Pearce about the melters’ first successful glass production and the challenges of vitrifying tons of nuclear waste. This interview was edited for length and clarity.

## Why did it take so long to get vitrification started at the plant?

It is the scale and the complexity of the waste that will soon be treated—much greater than that of Savannah River [a nuclear refinery site in South Carolina], where they have their own vitrification plant, which has been running for several years. The scale of the treatment plant here had to be much bigger. We often describe it as the most expensive chemical plant in the world.

## Issues were reported with the melters at Hanford in 2022. What happened?

The problem was related to that initial heating up of the melter and some of the heaters. They had to be replaced. Now they’ve got the new heaters functioning. They’ve melted the frit [granulated glass] and have poured the first test container, which is really exciting.

Right now it is just surrogate, nonradioactive glass-forming components that are relatively easy to form into a glass for this first test, before the radioactive waste is added, because nobody’s done anything like this on this scale before.

## What are the glasses made of?

The waste, rich in sodium and aluminium, is combined with glass-forming additives such as sand, boric acid, zircon, wollastonite, and olivine and heated until a glass is formed.

The glass has to have a certain heat capacity, viscosity, and durability. Initially, it’s a case of making a bunch of formulations and then testing them to make sure that they meet all these operational constraints before the glass can actually be made. You eventually end up with a matrix of compositions and an area of that matrix where you know the glasses will be durable.

## How do you test the glasses you are making for long-term stability?

Right now, they are validated by two kinds of short-term accelerated aging tests in the lab, but these tests are done at high temperatures, and that’s not representative of the disposal environment for the glass.

We’ve been trying to devise a test that will be more representative of those conditions using the U.S. Environmental Protection Agency’s LEAF [Leaching Environmental Assessment Framework], which allows you to do the test at room temperature and test things like liquid-to-solid ratio, because that’s really what drives the glass’s stability.

So you can do these shorter tests, but you really need to validate them by looking at glass that has been in the environment for 1,000-plus years. That’s where the analog work comes in. It’s challenging to find an analog that is close to the conditions of the IDF, so we did an excavation of the Broborg hillfort, in Knivsta, Sweden.

Broborg’s vitrified wall has really shown its durability in that near-surface environment for 1,500 years. I’ve got videos that show the archeologists going at that vitrified portion with crowbars and sledgehammers to try and break through it. That’s aluminosilicate glass, which is slightly different from the borosilicate glass we’re producing at the facility, but we didn’t start making borosilicate glasses until the last couple of hundred years, so there’s no ancient examples of that chemistry.

**Figure:**
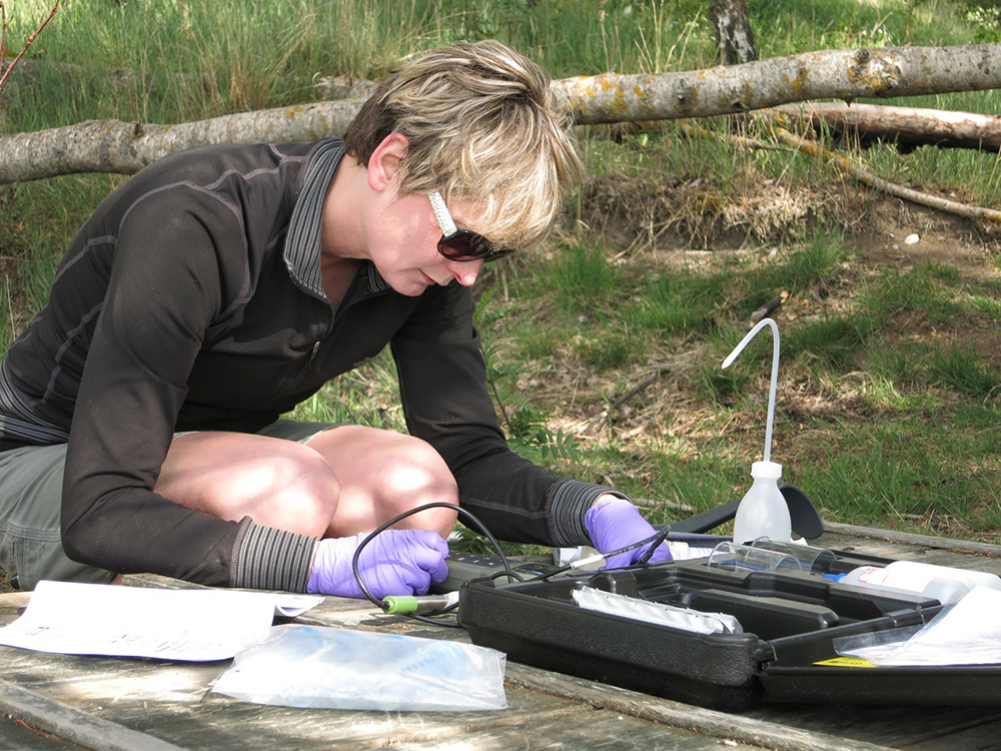
Carolyn Pearce takes pH measurements at Sweden’s 1,500-year-old Broborg hillfort. Credit: Rolf Sjöblom, Tekedo AB.

## What do you need to consider to create a stable glass composition for long-term storage?

For each tank, the chemistry is different. As that waste goes into the facility, it has to be characterized. Then the waste-processing team has to design the glass-forming components that will best immobilize that particular chemistry.

There’s a huge effort to determine the proportion of the glass that can be constituted from waste. We need to make sure that the mixture is stable over the planned time of performance, which right now is 1,000 years. We also want to make sure that the waste will retain the long-lived radionuclides and that once they hit the groundwater they will be released at such a rate that they stay below the drinking-water standards.

The challenge is actually things like aluminium, sodium, nitrate, hydroxide, and phosphate, because they’re in such high concentrations compared to the radioactive elements. For example, sodium hydroxide was added to waste to dissolve aluminium cladding that was on the uranium fuel rods. It was also added to the waste to raise the pH and inhibit corrosion of the carbon steel tanks.

The unpredictable behavior of these nonradioactive elements is a huge issue for efficiently processing the wastes. Aluminium is soluble in the highly concentrated sodium hydroxide solution that’s present, but it actually becomes supersaturated; if it precipitates in a pipeline, that can clog the pipes.

## What challenges remain unsolved in dealing with the waste at Hanford?

I think we’re pretty confident now that the plant that is processing low-activity wastes is going to work. Now, the attention is turning more toward the production and disposal of high-level waste. That is more challenging, because it is the sludge that is left behind once you remove all the supernatant. Sludge must be mobilized for transfer to the waste treatment plant, but sometimes it settles too fast posing a challenge to transport.

The sludge contains cesium and strontium, which are the shorter-half-life elements most responsible for the radioactive fields present in the tanks. But then you have these long-lived radionuclides like technetium, iodine, uranium, and some plutonium.

The technetium and the iodine are problematic because they are present in the waste as oxy anions, which are very soluble and do not really stick to anything. So if they were to be released, they would move quite quickly through soil into the groundwater.

## How does working in the national lab system differ from an academic lab?

The national lab environment is something that I’ve really enjoyed. You have to be able to work in teams and be very flexible, as you never know what the next need is going to be.

In my group, we work with seven partners, including national laboratories and universities. No one institution can do all the things that are needed to understand these complex problems.

## Rachel Brazil is a freelance contributor to

Chemical & Engineering News, *the independent news outlet of the American Chemical Society.*

